# Liposome encapsulated zoledronate favours M1-like behaviour in murine macrophages cultured with soluble factors from breast cancer cells

**DOI:** 10.1186/s12885-015-1005-7

**Published:** 2015-01-15

**Authors:** Sofia Sousa, Seppo Auriola, Jukka Mönkkönen, Jorma Määttä

**Affiliations:** 1 School of Pharmacy, Faculty of Health Sciences, University of Eastern Finland, Kuopio, Finland; 2 Institute of Biomedicine, Department of Cell Biology and Anatomy, University of Turku, Turku, Finland

**Keywords:** Breast Cancer, Tumour-associated Macrophages, Bisphosphonates, Clodronate, Zoledronate, Cytokines, Liposomes

## Abstract

**Background:**

Tumour stromal macrophages differentiate to tumour-associated macrophages (TAMs) with characteristics of immunosuppressive M2-type macrophages, having a central role in promoting tumour vascularisation, cancer cell dissemination and in suppressing anti-cancer immune responses. Bisphosphonates (BPs) are a group of drugs commonly used as anti-resorptive agents. Further, nitrogen containing BPs like Zoledronate (ZOL), are known to cause unspecific inflammatory reactions hence the hypothesis that its use could modulate TAMs polarization toward a more inflammatory phenotype.

**Methods:**

We studied the *in vitro* polarization of J774 murine macrophages upon culture in 4T1 breast cancer cell-conditioned medium (4T1CM) and stimulation with LPS and free and liposome-encapsulated bisphosphonates.

**Results:**

In this system, breast cancer soluble factors reduced the pro-inflammatory activation of macrophages but increased the secretion of matrix metalloproteinases (MMPs). In the presence of 4T1CM, a non-cytotoxic dose of liposome-encapsulated ZOL (ZOL-LIP) enhanced the expression of iNOS and TNF-α, markers of M1 activation, but did not diminish the expression of M2-type markers. In contrast, clodronate treatment either as a free drug (CLO) or liposome-encapsulated (CLO-LIP) decreased the expression of the M1-type markers and was highly cytotoxic to the macrophages.

**Conclusions:**

Breast cancer cells soluble factors modulate macrophages toward M2 activation state. Bisphosphonates may be applied to counteract this modulation. We propose that ZOL-LIP may be suitable for favouring cytotoxic immune responses by TAMs in breast cancer, whereas CLO-LIP may be appropriate for TAM depletion.

## Background

Macrophages exhibit a wide spectrum of activation phenotypes with two extremes (similar to the Th1/Th2 paradigm of T cells), the classically activated M1 macrophages and the alternatively activated M2 macrophages [1]. M1 macrophages protect against infections and tumour cell proliferation, phagocytose invading organisms, release nitric oxide (NO) or reactive oxygen species (ROS), present antigens to T cells and secrete immunomodulatory and pro-inflammatory cytokines [1,2]. M1 macrophages secrete low levels of IL-10 and high levels of IL-12, IL-6, and TNF-α and possess antitumour activity [2-6]. M2 macrophages scavenge tissue debris, display poor antigen presenting capabilities, promote angiogenesis and suppress T cell and natural killer cell proliferation and activity [7,8]. They produce high levels of IL-10, TGF-β, CCL-1, and CCL-22 and low levels of IL-12 and promote tumour growth and metastasis [3].

Tumour tissues are chronically inflamed and are capable of reprogramming the infiltrating immune cells to promote tumour progression [9,10]. The phenotype of most of the human tumour-associated macrophages (TAMs) resembles the M2 phenotype. These TAMs have been proven to support tumour growth, invasion, migration and metastasis [11].

Bisphosphonates (BPs) a class of anti-resorptive drugs are taken up by phagocytosing cells [12]. Based on their structure BPs can be divided into two categories, non-nitrogen-containing bisphosphonates (non N-BPs) and nitrogen-containing bisphosphonates (N-BPs). Both types have high affinity for bone [13].

Non N-BPs, such as clodronate (CLO), have a structure that closely resembles PPi and are incorporated into methylene-containing ATP analogues (AppCp-type metabolites). These ATP analogues are resistant to metabolism and inhibit cellular proliferation. They induce apoptosis by inhibiting the mitochondrial adenine nucleotide translocase (ANT) [13,14]. N-BPs, such as zoledronate (ZOL), inhibit farnesyl diphosphate synthase (FDPS), an enzyme of the mevalonate pathway. This leads to accumulation of unprenylated proteins and mevalonate pathway intermediates, such as isopentenyl pyrophosphate (IPP). IPP is further converted to triphosphoric acid 1-adenosin-5′-yl-ester3-(3-methylbut-3-enyl)ester (ApppI), possibly by aminoacyl-tRNA synthases [13,15]. ApppI also inhibits ANT thus inducing apoptosis. IPP is known to be an antigen recognised by γδ-T cells and such recognition, together with effects on monocytic lineage cells, contributes to the acute phase reaction, a common side effect of N-BPs [13,16].

N-BPs have been shown to inhibit macrophage proliferation, migration and invasion [17]. It has been demonstrated that N-BPs decrease MMP-9 secretion by breast tumour-bearing mice (the FVB x BALB-neuT model), thereby decreasing tumour infiltration by macrophages [18]. N-BPs have also been reported to reverse the polarity of TAMs from M2 to M1 in BALB-neuT and BALB-neuT/IFN-γ knockout murine models of mammary carcinoma [19]. TAMs are therefore highly suitable N-BPs target cells as long as their affinity for bone can be overcome *in vivo*. Liposomes have been proven to be effective carriers of BPs to macrophages enhancing BPs potency by factors of 20-1,000 [20].

In this study, we characterised for the first time the effects of soluble factors secreted by breast cancer cells on macrophage activation in the presence or absence of free and liposome-encapsulated BPs.

## Methods

### Liposome encapsulation of Zoledronate and Clodronate

Stock solutions of ZOL and CLO were encapsulated in negatively charged unilamellar liposomes by reverse-phase evaporation [20] using distearoylphosphatidylglycerol (DSPG) and cholesterol (2:1 ratio). The concentrations of ZOL and CLO were measured spectrophotometrically [20]. The lipid content of the liposomes was determined by a phosphorous assay [21] and the size distribution was analysed using the Nicomp 380XLS Zeta Potential/Particle Sizer, the mean diameter was under 300 nm. The concentration of the liposome-encapsulated compounds(X-LIP) was 1 mM ZOL-LIP and 10 mM CLO-LIP. The molar drug:phospholipid ratio was 1.5 for CLO-LIP and 0.5 for ZOL-LIP.

### Cell culture

With the exception of J774 cells, which were cultured in DMEM (Sigma), all other cell lines were cultured in RPMI-1640 (Sigma) supplemented with 10% FBS (Gibco) and 100 IU/ml penicillin and streptomycin (Gibco) at 37°C in a 5% CO_2_ atmosphere. The murine 4T1 breast cancer cell line and 3T3 fibroblast cell line were from the American Type Culture Collection. The J774 murine macrophage cell line was from the European Collection of Cell Cultures.

For studies of IPP, ApppI and AppCCl_2_p production and western blot analysis, cells were seeded into 6-well plates at a density of 5 × 10^5^ cells per well. Treatments were performed for 24 h, using 500 μM CLO, 10 μM ZOL, 50 μM CLO-LIP and 1 μM ZOL-LIP diluted in PBS.

### Cell viability, growth inhibition and cytotoxicity

To assess growth inhibition and cytotoxicity, J774 cells were plated at a density of 2.5 × 10^3^ and 1 × 10^4^ cells per well, respectively, in 96-well plates. Treatments were done with drug solutions from half-logarithmic dilution series, for 72 h. Cell viability was evaluated using the MTT assay as previously described [22].

### Studies assessing macrophage activation

Conditioned media (CM) from three independent 4T1 and 3T3 murine cell line batches were collected 72 h after the cells reached confluence. The batches were cleared by centrifugation and used as 50% supplements of J774 culture medium together with 10% FBS. After 72 h of culture with CM, J774 cells were stimulated for 24 h with 10 ng/mL LPS (*E. coli* serotype 026:B6, Sigma). LPS is a bacterial cell wall component known to act as a macrophage activator [23]. BPs were added 24 h before LPS stimulation (concentrations, see above). Cells were harvested for RNA extraction, and supernatants were collected for cytokine quantitation and Griess assay. Parallel FBS-free, LPS-treated supernatants were collected for zymography, and cells were harvested for acetonitrile (ACN)/water extraction and IPP, ApppI and AppCCl_2_p determination.

### HPLC-MS conditions for IPP, ApppI and AppCCl_2_p quantitation

IPP, ApppI and AppCCl_2_p were determined in dried ACN/water cell extracts by HPLC-ESI-MS as previously described [17,24]. Quantification of the molecules was performed using LCquan 2.0 software (Thermo Finnigan) using authentic standard curves with AppCp (Sigma) as an internal standard.

### SDS-PAGE and Western blot analysis

Whole cell lysates were prepared for SDS-PAGE and western blot analysis of FDPS (rabbit polyclonal anti-FDPS, Abgent)*,* Rap1A (goat polyclonal anti-Rap1A, Santa Cruz Biotechnology) and β-actin (mouse monoclonal anti-β-actin, Santa Cruz Biotechnology) as previously described [25]. An enhanced chemiluminescence (ECL) system was used for detection, and Image Quant RT ECL (GE Healthcare) was used for blot scanning.

### Cytokine quantification and Griess Assay

Interferon γ (IFN-γ), Interleukin 4 (IL-4), IL-10, IL-12(p70), IL-6, Macrophage Colony-Stimulating Factor (M-CSF), Monocyte Chemotactic Protein-1 (MCP-1), Tumour Necrosis Factor (TNF-α) and Vascular Endothelial Growth Factor (VEGF) were measured using a Murine Multiplex ELISA kit (Milliplex MAP-kit, Millipore, MCYTOMAG-70 K-9P) and analysed on a Luminex 200™ System. NO production was determined indirectly as nitrite (NO_2_^-^) content in culture supernatants using the Griess Reagent System (Promega).

### Zymography

The potential proteolytic activity of MMPs in the supernatants of treated J774 cells was determined by zymography as previously described [26]. The stained polyacrylamide gels were observed with Image Quant RT ECL. Densitometry of the bands corresponding to pro-MMP-9 activity (92 kDa) was performed using NIH ImageJ program.

### RNA analysis

RNA was extracted using the TRI Reagent (Applied Biosystems). RNA concentration was determined using NanoVue (GE Healthcare). cDNA was synthesised using the RevertAid kit (Fermentas).

Quantitative PCR (qPCR) primers were designed using Primer3 software [27] (Table 1). qPCRs were performed using the SYBR Green PCR Master Mix (Applied Biosystems) on an ABI Prism 7500 instrument (Applied Biosystems). Sequence-specific amplification of cDNAs was verified by melting-curve analyses. The threshold cycles (Ct) were normalised to the mRNA expression of endogenous GAPDH. Data analysis was performed using the Q-Gene program (Equation 2) [28].

**Table 1:** **List of primers used in the RNA analysis**

Transcript	Primers (5′ → 3′)	
iNOS	F	ACCGCACCCGAGATGGTCAGG
	R	TGCCGGCACCCAAACACCAA
IL-6	F	AGAGACTTCCATCCAGTTGCC
	R	TCTCATTTCCACGATTTCCC
IL-12	F	CCCCTGGAGAAACAGTGAACCT
	R	CACGTGAACCGTCCGGAGTA
MMP-9	F	CGGCACGCCTTGGTGTAGCA
	R	TCGCGTCCACTCGGGTAGGG
GAPDH	F	GCCGCCTGGAGAAACCTGCC
	R	GGGGTGGGTGGTCCAGGGTT

### Statistical analysis

All statistical analyses were performed using Prism 5 (Graphpad Software). Either a one-way ANOVA with Dunnett’s multiple comparison test (compared with the CTR) or an independent samples t-test with unequal variances (based on Levene’s test) assumption was used to analyse the significance of differences. P-values less than 0.05 were considered statistically significant.

## Results

### Soluble factors from breast cancer cells influence macrophage activation

**Figure 1:**
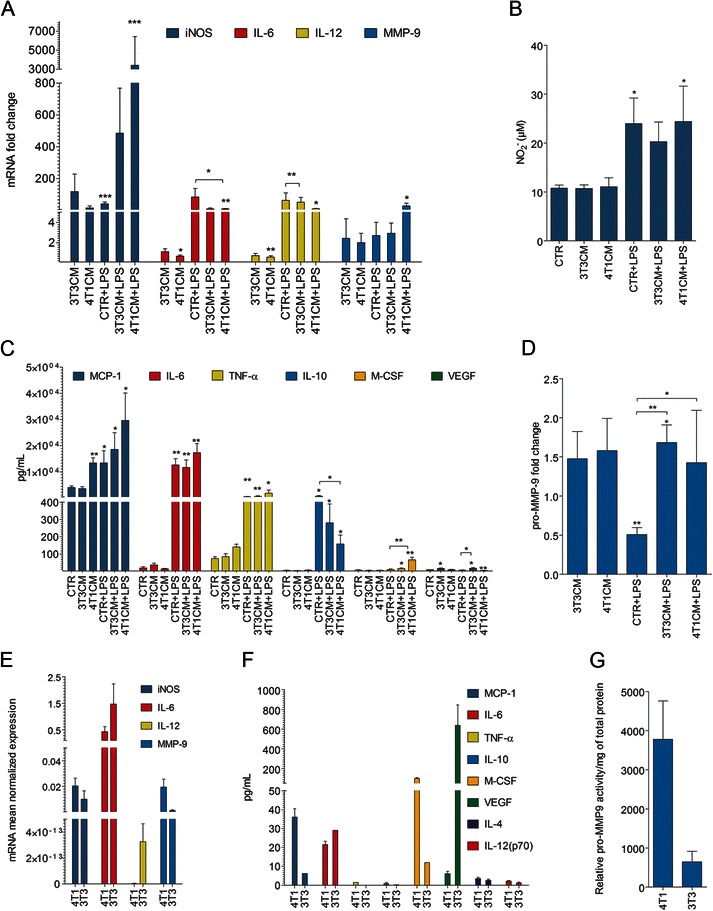
**Different mRNA and secreted cytokine profiles of 4T1 and 3T3 cells lead to differences in the modulation of J774 cell activation status with or without LPS stimulus. A** mRNA expression levels of iNOS, IL-6, IL-12, and MMP-9. **B** Protein levels of M-CSF, VEGF, TNF-α, IL-10, MCP-1 and IL-6 secreted by macrophages. **C** Nitrite (NO_2_^-^) production. Data represent the means ± SEM of 5 independent experiments. *p < 0.05, one-way ANOVA with Dunnett’s multiple comparison test as compared with the CTR. **D** Zymography analysis of pro-MMP-9 in cell supernatants . Data represent the means ± SEM of four independent experiments. *p < 0.05, **p < 0.005, student’s t-test as compared with the CTR or the CTR + LPS treatment (* lines above the bars). **E**-**G** Data represent the means ± SEM of 3 independent 72-h post-confluence cultures assayed in duplicate. **E** Mean mRNA expression levels of iNOS, IL-6, IL-12, and MMP-9 normalised to GAPDH. **F** Secreted protein levels of M-CSF, VEGF, TNF-α, IL-10, MCP-1 and IL-6. **G** Zymography analysis of pro-MMP-9 in cell supernatants.

The secretion of several enzymes and cytokines was affected upon macrophage culture with 3T3CM or 4T1CM. The secretion profile was especially influenced by the 4T1CM followed by LPS stimulus (Figure 1 and Table 2).

**Table 2:** **Summary of results section “Soluble factors from breast cancer cells influence macrophage activation”**

Cell lines		3T3	4T1	J774 Macrophages + CM				
Markers				3T3	4T1	LPS	3T3 LPS	4T1 LPS
M1	iNOS/NO_2_^-^	+++/ND*	+++/ND	≈CTR/≈CTR	≈CTR/≈CTR	>CTR/>CTR	≈CTR/≈CTR	**>CTR/>CTR** *
								**<LPS/≈LPS**
	TNF-α (protein)	-	**++**	≈CTR	≈CTR	>CTR	>CTR	>CTR
							≈LPS	≈LPS
	IL-6 (mRNA /protein)	+++/+++	+++/+++	≈CTR/≈CTR	**<CTR/≈CTR**	>CTR/>CTR		**>CTR/>CTR**
								**<LPS/≈LPS**
	IL-12 (mRNA /protein)	++/++	++/++	≈CTR/≈CTR	**<CTR/ND**	>CTR/ND	>CTR/ND	>CTR/ND
							≈LPS/ND	≈LPS/ND
M2	MMP-9 (mRNA /protein)	**+++/++**	**+++/+++**	≈CTR/≈CTR	≈CTR/≈CTR	≈CTR/<CTR	**>CTR/≈CTR**	**≈CTR/≈CTR**
							**≈LPS/>LPS**	**≈LPS/>LPS**
	VEGF (protein)	**+++**	**++**	**>CTR**	≈CTR	≈CTR	**>CTR**	**<CTR**
							**>LPS**	**≈LPS**
	IL-10 (protein)	**+**	**++**	≈CTR	≈CTR	>CTR	>CTR	**>CTR**
							≈LPS	**<LPS**
	M-CSF (protein)	**++**	**+++**	≈CTR	≈CTR	≈CTR	>CTR	**>CTR**
							≈LPS	**>LPS**
	MCP-1 (protein)	**++**	**+++**	≈CTR	**>CTR**	>CTR	**>CTR**	**>CTR**
							**≈LPS**	**≈LPS**

*ND, non detected; highlighted in bold are the significantly modulated markers either between the CM of 3T3 and 4T1 or compared to CTR or LPS macrophage cultures.

+, ++, +++ abundance of mRNA or protein.

The expression of the iNOS enzyme was increased upon LPS stimulation of macrophages and was further up-regulated upon incubation with 4T1CM followed by LPS (Figure 1A). The increased expression of iNOS always correlated with an increase in NO secretion (Figure 1C).

The expression and secretion of IL-6 and IL-12 by macrophages was different if the cells were treated with 4T1CM alone or together with LPS (Figure 1A,B). 4T1CM significantly decreased the mRNA expression of these cytokines (IL-6, 0.7 and IL-12, 0.6-fold change, p = 0.006 and p = 0.007, respectively). Treatment with 4T1CM followed by LPS yielded a 10-fold increase in the mRNA expression of both IL-6 and IL-12 (p = 0.003 and p = 0.017, respectively). Further, 4T1CM reduced IL-6 mRNA expression upon LPS stimulation when compared with LPS treatment alone (p = 0.041). The control 3T3CM induced no such differences, suggesting cell line specificity of the modulating factors (Figure 1A).

The secretion of IL-6 protein by macrophages reflected the mRNA expression profile, except when the cells were treated with 4T1CM followed by LPS; here, the decrease observed at the mRNA level was not observed at the protein level (Figure 1A,B). Treatment with 3T3CM and 4T1CM followed by LPS led to a significant increase in M-CSF secretion when compared with unstimulated macrophages (p = 0.027 and p = 0.005, respectively). Furthermore, incubation with 4T1CM, but not 3T3CM, significantly increased the LPS-mediated M-CSF secretion by macrophages (p = 0.007).

Secretion of VEGF by macrophages was significantly increased only upon 3T3CM treatment alone or followed by LPS stimulation. In the latter conditions, VEGF secretion was even higher than that induced by LPS treatment alone. Incubation with 4T1CM together with LPS decreased VEGF production by macrophages when compared with control-treated cells (Figure 1B).

Secretion of TNF-α and IL-10 was enhanced by treatment with LPS (Figure 1B). This increase in IL-10 secretion was significantly (p = 0.039) lower when the macrophages were pre-treated with 4T1CM.

Incubation of macrophages with both 4T1CM and LPS had similar agonistic but not additive effects on MCP-1 secretion. Pre-treatment of macrophages with 3T3CM prior to LPS stimulation resulted in a 2.5-fold increase in the mRNA expression of MMP-9 and unique 1.5-fold increase in MMP-9 enzymatic activity (Figure 1D). The level of secreted MCP-1 protein directly correlated with the levels of secreted M-CSF and TNF-α. IL-4 and IL-6 protein levels inversely correlated with M-CSF and TNF-α levels (r = ±0.943 and p = 0.017, respectively, Spearman nonparametric correlation).

### Secretion profile of breast cancer cells and fibroblasts

4T1 and 3T3 cells had similar IL-6 and IL-12 mRNA and protein levels (Figure 1E and F, respectively).

The amount of IL-10 secreted by 4T1 cells was low, but higher than that produced by 3T3 cells (Figure 1F). Both cell lines secreted very low amounts of IL-4 (Figure 1F). The presence of IL-4 and IL-12(p70) was detected but at levels too low to reliably quantify.

4T1 and 3T3 cells secreted M-CSF (99.38 ± 8.94 pg/mL and 11.95 ± 6.19 pg/mL) and MCP-1 (36.03 ± 6.29 pg/mL and 6.20 ± 2.13 pg/mL), respectively (Figure 1F).3T3 cells released considerable amounts of VEGF protein (638.47 ± 291.11 pg/mL), in contrast to 4T1 cells (Figure 1F). Only 4T1 cells secreted detectable, but low, amounts of TNF-α (Figure 1F). The relative mRNA expression of iNOS and MMP-9 was similar between the two cell lines. (Figure 1E). MMP-9 proteolytic activity was higher in 4T1 than in 3T3 cells (Figure 1G).

### Effect of free and liposome-encapsulated BPs on macrophages

**Figure 2:**
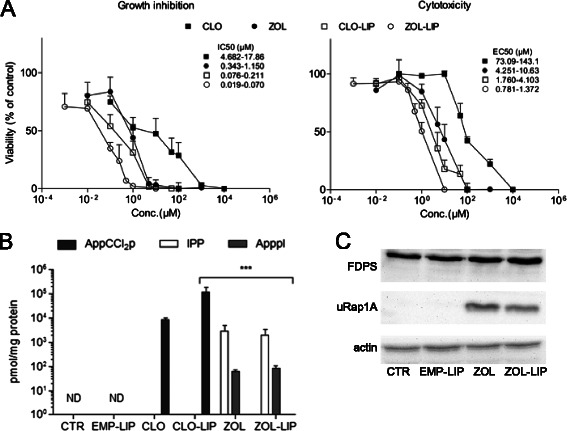
**Comparison of free and liposome encapsulated CLO and ZOL potencies. A** Effect of BPs on the growth inhibition and cytotoxicity of J774 cells after 48 h of treatment. IC50 and EC50 values represent the 95% confidence intervals of the normalised, non-linear fitted values. **B** IPP, ApppI and AppCCl_2_p accumulation in J774 cells after BP treatment. ND, levels below the limit of detection. ***p < 0.0001, one-way ANOVA with Dunnett’s multiple comparison test as compared with the CTR. **C** Western blot analysis of the effect of ZOL, ZOL-LIP and EMP-LIP treatment on protein prenylation and other key mevalonate pathway molecules. The results are representative of three independent experiments.

ZOL was approximately 10-fold more potent than CLO both in inhibiting growth and in inducing cell death in J774 macrophages (Figure 2A). Liposome encapsulation led to approximately 100-fold and 10-fold increase in those capabilities of CLO and ZOL, respectively. As demonstrated previously [29], treatment with CLO led to the accumulation of the ATP analogue, 5′(β,γ-dichloromethylene)triphosphate (AppCCl_2_p) (Figure 2B). Like the free drug [17], ZOL-LIP inhibited FDPS, resulting in the accumulation of IPP, ApppI and unprenylated Rap1A (uRap1A) (Figure 2B,C).

**Figure 3:**
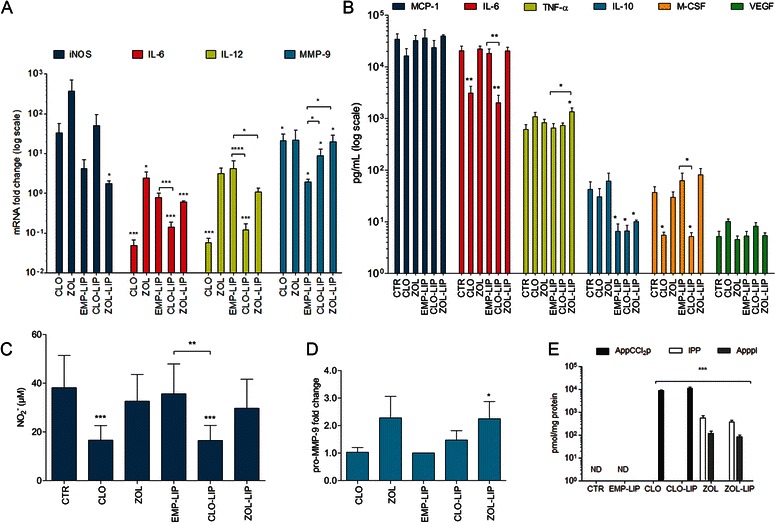
**Effect of BPs on the response to LPS in 4T1CM-treated J774 cells. A** mRNA expression of iNOS, IL-6, IL-12, and MMP-9. **B** Protein levels of M-CSF, VEGF, TNF-α, IL-10, MCP-1 and IL-6 secreted by macrophages. *p < 0.05, **p < 0.005, student’s t-test as compared with the CTR and EMP-LIP (* lines above the bars). **C** Nitrite (NO_2_^-^) production. *p < 0.05, one-way ANOVA with Dunnett’s multiple comparison test as compared with the CTR. **A**-**C** Data represent the means ± SEM of 4 independent experiments. **D** Zymography analysis of pro-MMP-9 in J774 cell supernatants. Presented values are the optical densities of pro-MMP-9-digested bands normalised to the total protein content of the corresponding total cell lysate compared with appropriate controls (CTR or EMP-LIP). **E** IPP, ApppI and AppCCl_2_p levels after treatment. Data represent the means ± SEM of three independent experiments. ***p < 0.0001, one-way ANOVA with Dunnett’s multiple comparison test as compared with the CTR.

### CLO and ZOL have different effects on the activation status of 4T1CM-primed macrophages

ZOL-LIP treatment induced a significant (2-fold) increase in iNOS expression (Figure 3A), but not in NO secretion by J774 cells (Figure 3C). Treatment with CLO and CLO-LIP resulted in a significant decrease in NO production (Figure 3C), but this was not observed at the mRNA level (Figure 3A).

CLO, CLO-LIP and ZOL-LIP treatments significantly decreased the mRNA expression of IL-6 and IL-12 in J774 cells (Figure 3A). IL-6 protein levels were also significantly decreased upon CLO and CLO-LIP treatment (p = 0.006 and p = 0.005, respectively) (Figure 3B). At the mRNA level, treatment with both CLO and CLO-LIP increased MMP-9 expression in J774 macrophages (Figure 3A). However, the mRNA data were not reflected in the enzymatic activity as determined by zymography (Figure 3D). Only the ZOL-LIP treatment induced an over 10-fold increase in MMP-9 mRNA expression and a 2-fold increase in enzymatic activity (Figure 3A,D).

Incubation with CLO and CLO-LIP decreased M-CSF secretion by macrophages (p = 0.016), whereas incubation with ZOL and ZOL-LIP had no effect (Figure 3B). All the liposomal treatments, even empty liposomes (EMP-LIP), decreased the protein secretion of IL-10, suggesting an effect of the liposomal formulation *per se* rather than a BPs’ effect (Figure 3B). ZOL-LIP was the only treatment that significantly increased TNF-α secretion by macrophages when compared to untreated or EMP-LIP treated cells (p = 0.017 and 0.021, respectively; Figure 3B). 4T1CM together with LPS stimulation did not significantly affect the BP induced accumulation of AppCCl_2_p, IPP or ApppI in macrophages (Figure 3E), indicating that the mechanisms of BPs action were not affected [13].

Secreted MCP-1 protein levels correlated with M-CSF protein levels (r = 0.943, p = 0.017), and IL-6 protein levels inversely correlated with VEGF protein levels (r = -0.886, p = 0.03) (Spearman nonparametric correlation). In all the conditions tested (Figures 1C and 3B), IFN-γ levels were below the limit of detection.

## Discussion

Incubation of J774 macrophages with 4T1CM reduced the mRNA expression of IL-6 and IL-12 and made them less responsive to LPS. These findings suggested that 4T1CM diminished the capability of macrophages to mount a pro-inflammatory response. However, there appeared to be a delay between the transcriptional and secretory IL-6 responses. In the multiplex ELISA we analyse only the secreted cytokines in the macrophage culture supernatants and not total levels, the mismatched mRNA and protein levels may therefore be a combination of miRNA translational block of IL-6 mRNA or a decreased secretion of the produced protein, especially as the expected result was a decrease from rather high IL-6 secretion induced by the LPS stimulus. Cytokines in the supernatants and mRNAs were analysed at the same time point, in matched samples (i.e. supernatant was collected from the same well used for RNA extraction).

4T1CM treatment counteracted the LPS-induced decrease in MMP-9 activity, suggesting that 4T1CM increased MMP-9 expression and that the activity of MMP-9 was not fully suppressed by LPS. The soluble factors secreted by 4T1 cells appeared to reduce the production of inflammatory cytokines and favour the expression of MMP-9. The cytokine expression profile of the 4T1CM-treated macrophages was mostly characteristic of M2-type cells. However, there was still an increased iNOS expression and NO production, markers typical of an M1 phenotype [30]. Nevertheless, several studies have indicated a dual time and concentration dependent role for iNOS expression in breast cancer progression. Mice lacking iNOS experience longer periods of latency in mammary tumour growth. In contrast, high NO production promotes the cytotoxicity of TAMs against breast cancer cells, and low NO concentration promotes angiogenesis and cancer cell invasion. In a co-culture study breast cancer cells induced NO production by macrophages, which promoted cancer cell invasion favouring VEGF-A and MMP-9 expression [31,32].

Treatment with both 3T3CM and 4T1CM sensitised macrophages to LPS in terms of M-CSF production. The two cell lines produced significant amounts of M-CSF, especially 4T1. The higher M-CSF protein levels observed in the supernatants of macrophages treated with 4T1CM and LPS might be the sum of macrophage- and 4T1 cell-derived M-CSF. In the context of breast cancer metastasis to bone, it is known that the M-CSF produced by human breast cancer cell lines increases osteoclast survival and activity [33], stimulates macrophage expansion, and up-regulates receptor activator of nuclear factor kappa-B ligand (RANKL) expression in stromal cells [34]. Furthermore, binding of breast cancer cell-derived M-CSF promotes epithelial growth factor (EGF) production by macrophages. This process leads to the reciprocal activation of invasion and co-migration of both cell types [35]. These observations were supported by our results. M-CSF was secreted by macrophages incubated in 4T1CM, and the secretion was boosted with LPS simulus.

MCP-1 was produced by 3T3 cells and at higher levels by 4T1 cells. A positive feedback loop has been demonstrated in mouse mammary tumours whereby the cancer cells release MCP-1; this promotes macrophage secretion of TNF-α, which in turn promotes further MCP-1 expression by cancer cells [36]. A similar loop was observed in the present study, as 4T1CM pre-treated macrophages demonstrated an elevated secretion of both MCP-1 and TNF-α.

4T1CM alone did not affect TNF-α secretion by macrophages, but the media had an agonistic effect on the cells when LPS was introduced. Similar results have been demonstrated in a human co-culture study in which cancer cells increased TNF-α expression in macrophages, thus inducing MMP expression and activity [37]. In the present study, both fibroblast- and breast cancer-CM showed similar effects on macrophages, as both induced higher secretion levels of TNF-α and higher MMP-9 activity in LPS stimulated macrophages.

Cancer cells produce small amounts of VEGF, a protein that could auto-stimulate neoangiogenesis and tumour growth. However, fibroblasts and macrophages produce considerably larger amounts of VEGF being in that sense more significant supporters of tumour growth rather than the cancer cells alone [38]. In a co-culture model, breast cancer cell secreted factors increased VEGF production by macrophages [38]. In our study, 3T3CM was the strongest stimulus inducing VEGF secretion by macrophages, a result emphasising the relevance of fibroblast-secreted factors in cancer progression. The VEGF levels may be attributed to both macrophage and 3T3 cell production.

Release of IL-10 is a well-described monocyte response to LPS. However, in human PBMCs, IL-10 pre-treatment has been shown to desensitise the cells to LPS in terms of IL-10 and TNF-α production [39]. The same phenomenon may explain the diminished IL-10 response observed in the LPS stimulated macrophages pre-treated with 4T1CM, as 4T1 cells secreted more IL-10 than 3T3 cells.

As previously demonstrated, treatment with both CLO and CLO-LIP decreased the NO, IL-6, and IL-12 mRNA and protein expression in macrophages [40], and for the first time, we showed decreased M-CSF mRNA and protein production by CLO and CLO-LIP treated macrophages. The effect of CLO alone was a real transcriptional effect, as cell viability was not affected (results not shown). CLO-LIP treatment reduced cell viability. Thus, its effect may be partially attributed to cytotoxicity. Similarly to reports using other N-BPs, ZOL treatment did not have an effect on macrophage NO production [41,42]. IL-6, IL-12, cytokines related to the acute phase reaction, are known to be up-regulated by low-dose N-BPs both *in vivo* and *in vitro* [40,41,43,44]. In studies with alendronate, the acute phase cytokine profile has been shown to be unrelated to FDPS inhibition [44]. However, no previous *in vitro* data exist for the effects of ZOL or ZOL-LIP in this context. Our results further confirmed that N-BPs interfere with macrophage cytokine production, but the mechanism of action of this effect is still unknown. ZOL treatment appeared to increase the mRNA expression of IL-6 and IL-12, but this increase was not detected at the protein level. ZOL-LIP treatment decreased IL-6 mRNA similarly as previously reported for liposome-encapsulated pamidronate (an N-BP) and CLO-LIP [40].

The increase in TNF-α production observed upon exposure of macrophages to ZOL-LIP is consistent with the effect of pamidronate [45], which induced TNF-α production by mouse macrophages. In the same study, it was shown that CLO suppressed IFN-γ-induced TNF-α production [45]. The N-BP-induced increase in TNF-α production has been observed also in cultured human PBMCs treated with pamidronate [46]. We have previously shown the opposite effects of N-BPs and non-N-BPs on TNF-α production by macrophages [47]. In the current study, CLO and CLO-LIP treatments did not significantly decrease TNF-α secretion by macrophages, whereas a significant increase was observed upon exposure to ZOL-LIP. Activation of TNF-α production by N-BPs may be beneficial, as certain malignant tumours are sensitive to this cytokine [48].

The apparent discrepancies between MMP-9 mRNA expression and its potential activity levels are consistent with a previous study on the effects of BPs on MMPs [49]. Further, it has been demonstrated that CLO inhibits the enzymatic activity of purified MMP-9 protein, whereas ZOL demonstrates no such inhibition [50]. In our study, both CLO and CLO-LIP treatments enhanced the mRNA expression of MMP-9, whereas only ZOL-LIP treatment increased both MMP-9 expression and activity. Our results with ZOL-LIP treatment confirmed previous findings where ZOL did not inhibit MMP-9 activity [50]. The ZOL doses used in the current study are comparable to the low pamidronate doses used by others [49]. The increased MMP-9 expression therefore appears to be a typical response to low-dose N-BP treatments. Higher doses of N-BPs inhibit protein prenylation, thereby impairing several cellular functions, and they most likely also diminish MMP enzyme production or secretion. Recently, exogenous gene transfer of MMP-9 was shown to suppress tumours by increasing neutrophil infiltration into the tumour site, a process that enhances macrophage secretion of pro-inflammatory cytokines and decreases IL-10 production [51]. In light of this, enhanced MMP-9 expression by macrophages upon exposure to ZOL-LIP may be beneficial in the tumour microenvironment.

## Conclusions

To our knowledge, the above effects have never been shown in murine macrophage cell lines conditioned exclusively with soluble factors secreted by murine breast cancer cells. The findings are in line with those from similar studies performed with freshly isolated human monocytes and conditioned media from melanoma cells [3]. Additionally, the current findings are of particular relevance because the cross talk between human macrophages and breast tumour cells is essential for the first steps in metastasis formation [52].

Our results suggest that the effects of non-N-BPs and N-BPs on macrophage activation are distinct, when used in sub-lethal doses. If the objective is to reduce inflammation or to eradicate macrophages, a non-N-BP may be used, as we saw that CLO and CLO-LIP were cytotoxic to macrophages and decreased most of the M1 markers tested. If the aim is to alter macrophage activation without decreasing inflammation, an N-BP may be more adequate, as it was seen that in the doses used ZOL-LIP presented few cytotoxic effects and maintained (NO_2_^-^, IL-6, IL-12) or increased(iNOS and TNF-α) M1 markers after LPS activation.

## Abbreviations

ACN: Acetonitrile
ANT: Adenine nucleotide translocase
AppCCl_2_p: 5′(β,γ-dichloromethylene)triphosphate
ApppI: Triphosphoric acid 1-adenosin-5′-yl ester 3-(3-methylbut-3-enyl) ester)
BP: Bisphosphonate
CLO: Clodronate
CLO-LIP: Liposome-encapsulated CLO
CM: Conditioned media
EGF: Epithelial Growth Factor
EMP-LIP: Empty liposome
FDPS: Farnesyl diphosphate synthase
GAPDH: Glyceraldehyde 3-phosphate dehydrogenase
IFN-γ: Interferon γ
IL: Interleukin
iNOS: Inducible nitric oxide synthase
IPP: Isopentenyl pyrophosphate
LPS: Lipopolysaccharide
MCP-1: Monocyte chemotactic protein-1
M-CSF: Macrophage colony-stimulating factor
MMP: Matrix metalloproteinase
N-BP: Nitrogen-containing BP
NO: Nitric oxide
non-N-BP: Non-nitrogen-containing BP
PBMCs: Peripheral blood monocytes
RANKL: Receptor activator of nuclear factor kappa-B ligand
TAM: Tumour-associated macrophage
TNF-α: Tumour necrosis factor α
uRap1A: Unprenylated Rap1A
VEGF: Vascular endothelial growth factor
ZOL: Zoledronate
ZOL-LIP: Liposome-encapsulated ZOL
